# Thailand Momentum on Policy and Practice in Local Legislation on Dengue Vector Control

**DOI:** 10.1155/2014/217237

**Published:** 2014-04-01

**Authors:** Adisak Bhumiratana, Apiradee Intarapuk, Suriyo Chujun, Wuthichai Kaewwaen, Prapa Sorosjinda-Nunthawarasilp, Surachart Koyadun

**Affiliations:** ^1^Department of Parasitology and Entomology, Faculty of Public Health, Mahidol University, 420/1 Rajvithi Road, Rajthewee, Bangkok 10400, Thailand; ^2^Department of Clinic, Faculty of Veterinary Medicine, Mahanakorn University of Technology, 140 Cheum-Sampan Road, Nong-Chok, Bangkok 10530, Thailand; ^3^Ministry of Public Health, Department of Disease Control, Office of Disease Prevention and Control 11, Nakhon Si Thammarat 80000, Thailand; ^4^Department of Geoinformatics, Faculty of Geoinformatics, Burapha University, Chonburi 20131, Thailand; ^5^Department of Fundamentals of Public Health, Faculty of Public Health, Burapha University, Chonburi 20131, Thailand

## Abstract

Over a past decade, an administrative decentralization model, adopted for local administration development in Thailand, is replacing the prior centralized (top-down) command system. The change offers challenges to local governmental agencies and other public health agencies at all the ministerial, regional, and provincial levels. A public health regulatory and legislative framework for dengue vector control by local governmental agencies is a national topic of interest because dengue control program has been integrated into healthcare services at the provincial level and also has been given priority in health plans of local governmental agencies. The enabling environments of local administrations are unique, so this critical review focuses on the authority of local governmental agencies responsible for disease prevention and control and on the functioning of local legislation with respect to dengue vector control and practices.

## 1. Introduction

Singapore is one of the Southeast Asian countries that had implemented the successful dengue vector control program since 1973 by making use of 3 principal strategies, for example, source reduction of* Aedes aegypti* population, public education, and law enforcement [1]. As to stop propagation of* Ae. aegypti *in human habitations, the Destruction of Disease Bearing Insects Act (1968) was enacted [1] and, consequently, dengue had been contained, but not domestically arrested, by a 15-year period of low dengue incidence. However, dengue reemerged since the 1990s despite stability of low level of infestation. Law enforcement alone is not a mainstay of strategy used in effective and sustained dengue vector control [1, 2]. However, this is the challenge for Thailand that has been moving toward decentralization; the implementation of local administration has offered public health challenges (e.g., healthcare services, disease prevention/control, and local legislation and regulation of dengue vector control) to both local governmental agencies and other public health agencies at all ministerial, regional, and provincial levels [3]. Understanding the multiple facets of administrative decentralization model adopted for local administration development in Thailand is fundamental to comprehend the direct and indirect impacts on dengue vector control including enforcement of regulations by local governmental agencies across the country. Thus, based on the evaluation of local legislation and regulation on dengue vector control between 2011 and 2012, this paper addresses 3 aspects of the argument: authority of local administration organizations, legislation on dengue vector control, and recommended good practice for local administrators.

## 2. Authority of Local Administration Organizations

### 2.1. Policy and Direction of Local Administration Development

Thailand is a unitary state that has 76 provinces as administrative divisions and the capital Bangkok as a unique administrative division governed by the elected mayor and board. The province is divided into three different levels of administrative subdivisions (i.e., district, subdistrict, and village). The country has been moving toward decentralization since the early 1990s [3]. The state policy on decentralization has increasingly encouraged the development of local administration at provincial level (Table 1). Regarding this, the province has three different levels of municipalities (i.e., city, town, and subdistrict municipality) and also has two different levels of local administrative organizations (LAOs) (i.e., provincial and subdistrict), all of which are administered by the elected mayor and board. On the other hand, the implementation of complex bureaucracy of local administration has offered challenges to the provincial and subdistrict levels of LAOs as well as the municipalities because these local governmental agencies have jurisdiction as multifunction agencies that play key roles in decentralization in the boarder context of local administration development (Table 1) [3–6]. All levels of local governmental agencies or LAOs are specifically responsible for the administration and finance, delivering public services, and, more importantly, encouraging public participation. Therefore, they are the integral parts of provincial strategic development of socioeconomy as well as human well-being and universal health care across the country.

### 2.2. Local Authority of Disease Prevention and Control

In the context of public health, the decentralization has also impacts on the structural organization, authorization, and responsibilities of the responsible public sectors—including the Ministry of Public Health (MoPH) as national health authority, the Provincial Public Health Offices (PPHOs) as provincial health authority, and the LAOs as local authority. The MoPH and PPHOs play pivotal roles as partners or enterprising counterparts of LAOs—(1) they provide the public health policy advocacy and direction, (2) they support comprehensive health system management radically upon the equity of universal access to health care services to local people, (3) they mobilize resources available for that health system management, and (4) they set and monitor the standards and quality control of healthcare services. Given their power and duty on public services, the LAOs are expected to take into account the implementation of interventions and services for disease-oriented public health programs through their strategic plans and funded projects for rural or urban health development.

In fact, only the SAOs and municipalities have the authority of disease prevention and control as the integral part of public services after provisions of the laws (Table 2). However, disease surveillance—that governs routine diagnosis, background surveillance and reporting, identification of disease prevalence or incidence with demographic/geographic differentiation and trend, epidemiologic investigation, and epidemic forecast—is not the duty of the SAOs and municipalities. In Table 2, the authority of disease prevention and control for only the SAOs has been stipulated after provisions of the laws relating to local administration, decentralization, and public health. Over the past decade, the SAOs as well as the municipalities have been less likely to practice LDPC, but the public health agencies are more likely to issue a general consideration of local public health regulation and legislation regarding disease prevention and control in the administration arm of these LAOs. For example, more than 5000 SAOs across the country (Table 1) have been responsible for the size and scope of public health problems including dengue [3] such that disease prevention and control, but not surveillance for notifiable diseases, need to be warranted. Thus, the question is raised about how local Thai people, geographically confined to different SAO settings, participate in decision-making and monitoring of disease prevention/control activities implemented by the SAOs and what mechanism is augmented to strengthen their capacity of disease prevention and control.

## 3. Legislation on Dengue Vector Control

As mentioned earlier, dengue has become a major public health problem affecting local Thai people across the country [3]. Dengue prevention and control have been integrated into healthcare services at the provincial level, but this offers the challenge to the SAOs and municipalities as the certain situations on the risks for dengue transmission require the management along with special expertise to leverage needed data/information of the infestation and reinfestation of* Aedes* vectors, to implement dengue vector control measures and activities efficiently and effectively [3] or to establish well-functioning services of integrated vector management (IVM) for other vector-borne diseases and pest control [7, 8].

Typical dengue vector control focuses on preventing dengue vectors that infest water-holding containers. This is an inexpensive and simple expedient, but there seems to be synergism of “top-down” policy and conscientious execution, especially in determining leadership and partnership, and “bottom-up” practices in household-level environmental management [3, 6, 9–14]. In regard to the vertical dengue vector control alone or IVM practices in Thailand, LAOs including SAOs may employ a local legislation, but they do not exploit its enactment as a powerful tool for effective and sustained environmental management [3, 8]. The legitimate local governments should interest any community stakeholder in an enterprise. However, the LDVC and practices—targeting rural settings by the SAOs—may differ from that executed by the municipalities—targeting urban settings. This is because the environment of local administration is dependent on the force of the circumstances to which the sociopolitical, economical, and cultural components are related. Only the LDVC and proactive actions by the SAO are discussed below in details.

### 3.1. Delegated Legislation of SAO: Rising Obligation, Melting Firm

Assigned as the regulator, the SAO interplays with other local health authorities in reduction of the ultimate consequences of health risks associated with the endemic transmission of dengue or with the outbreaks or epidemics of dengue fever or dengue hemorrhagic fever [3]. As for the local legislation and regulation, most contract SAOs (i.e., to contract the arrangement of local code on dengue vector control) authorize both legislative proceedings through a legislative body and proclamation of the dengue vector control. The practice of LDVC is dependent on the delegated powers and duties—issued by the section numbers 67, 71, and 73 of the Part 3 Authority, Chapter 2 of the Subdistrict Administrative Organization of the Subdistrict Council and Subdistrict Administrative Organization Act (1994) and the following regulations: (1) the Constitution of the Kingdom of Thailand (2007) and the Official Information Act (1997) and (2) the regulatory sections of the Public Health Act (1992) and the Notification of the Abatement of* Aedes* Breeding Place (2002) (Table 2). In this regard, a local legislative body comprises the elected mayor, board, and council of the SAO and a district chief; all of them are in charge of the local legislation including dengue vector control code.

Nonetheless, almost all contract SAOs—whether they hold a proclaimed authoritative statement of the local code of abatement of* Aedes* breeding place—applied the context of dengue vector control by determining additional terms or by amending the meaning of terms, as discussed below. Why does the written statement of this local ordinance differ from that of the notification of abatement of* Aedes *breeding place issued by the Minister of Public Health? In fact, the challenge is that the SAOs must rely on principles applicable in this local ordinance to cope with dengue transmission dynamic underlying geographical differentiation and vector biology [9, 15–17], especially insecticide resistance in local* Ae. aegypti* [18]. Based on the evaluation of local legislation on dengue vector control across the country between 2011 and 2012, the authors elaborate two articulate issues on the significance of the notification of abatement of* Aedes *breeding place and the transaction of the local code of abatement of* Aedes *breeding place. The critics have disparate ideas on how and what strategy can reduce any misuse or misconduct, while it can increase the acceptance and compliance of that local code.

### 3.2. Significance of the Notification of Abatement of* Aedes* Breeding Place

As to the sections 5 and 25 (paragraph 5) of Chapter 5 Sources of Nuisance of the Public Health Act (1992), the Minister of Public Health issued the Notification of Abatement of* Aedes* Breeding Place as a Source of Nuisance (2002) to the public audiences. The notification issues the matter of* Aedes* breeding place as a source of nuisance, and public health officials are assigned to operate the abatement of* Aedes* breeding place, as described below. As a matter of fact, there is no determination of punishment in case of misuse or misconduct. This notification became effective after the Minister officially proclaimed in the Royal Thai Government Gazette on 25th June, 2002, during which the epidemic dengue had occurred and spread across the country [3].

“*Minister of Public Health shall be in charge of this Act, authorize the assignment to public health officials, issue ministerial regulation on a fixed fee or an exempt fee, and prescribe others required to execute this Act. The ministerial regulation shall become effective soon after the promulgation in the Royal Thai Government Gazette”*, regarded as the section 5 of the Public Health Act (1992).

*“In the event that it might exasperate neighbors or other exposed persons, it shall be a source of nuisance.*

*(5) Any other source prescribed by the Minister and promulgated in the Royal Thai Government Gazette”*, regarded as the section 25 (paragraph 5) of the Public Health Act (1992).

As with the Public Health Act and its amendment issue and notification, the regulators or authorized officials whom are appointed to execute this Act include both public health and local officials as follows.

*“A public health technical officer who holds current position of infection control technical officer, practitioner level-5 or above, and belongs to the Section of General Communicable Diseases, the Cluster of Technical Promotion and Health Care Services, Provincial Public Health Office shall be assigned as public health official as issued by the Public Health Act, B.E. 2535”*, regarded as the portion 3 of the Notification of Abatement of* Aedes* Breeding Place (2002).

*“Local official means the mayor of Subdistrict Administrative Organization”*, regarded as the section 5 of the Amendment Issue No. 2 of the Public Health Act (2007).

Public health official, but not local official, is assigned by the Minister of Public Health. In practice, a public health official is assigned by the PPHO, while a local official is assigned by the SAO. These authorized officials or regulators have the authority in the obligatory implementation of dengue vector control and other prescribed methods. The MoPH has urged the regulators from all public sectors to be responsible for the containment of environments favorable to the breeding of* Aedes* vector [3].

As for the section 25 (paragraph 1) of Chapter 5 Sources of Nuisance and the section 4 of the Public Health Act (2002), the descriptors “*breeding place for disease vector*” and “*buildings*” are pivotal for the provision of the Notification of Abatement of* Aedes *Breeding Place.

*“In the event that it might exasperate neighbors or other exposed persons, it shall be a source of nuisance.*

*(1) Water source, water drainage, bathroom, latrine, pit latrine, ash pit, or any other place in impoverished area in which its environment remains unclean, accumulated, or filled with wastes. This causes noisome matters or toxic particles; or this becomes or seems to be a breeding place for disease vector, to impair one's health, or to harm one's health”*, regarded as the section 25 (paragraph 1) of the Public Health Act (2002).

*“Buildings mean any houses, plants, shops, warehouses, offices, or any other buildings accessible for the people likely to stay or utilize”*, regarded as the section 4 of the Public Health Act (2002).

With respect to the portion 1 of the Notification of Abatement of* Aedes* Breeding Place, four descriptors that equate the environments favorable to the breeding of* Aedes* vectors are therefore buildings, water-holding containers, discarded receptacles, and* Aedes *breeding places. These descriptors are solemnly considered the sources of nuisance, as discussed below. Because* Ae. aegypti* is potentially adapted to its local environments close to human habitations, the peri-domestic* Ae. aegypti* rather than* Ae. albopictus *becomes the primary dengue vector susceptible to any dengue serotypes and responsible for dengue transmission [3, 9, 15, 16]. However, the issues on public spaces and waste disposal management are not articulated.

“*Buildings*” refer to any construction with specific purposes of use. As for human habitation, an enclosed permanent or temporary structure with a roof and other compartments including the walls, ceilings, floors, and other attached amenities and fixtures can create favorable environment and human-vector contact site at which anthropophagous and endophagous* Ae. aegypti* forages any human blood meal during daytime and then breeds their progeny in any potential water-holding container or discarded receptacle, and vice versa. Other than human habitation, buildings used for manufacturing, trading, transportation, and other public service activities are also considered the contact site. Healthy houses and environments become basically important for a primary prevention of dengue to reduce human-vector contact. More obviously, there is controversy that human-*Aedes* contact site relates to housing construction, surrounding environments, or both [3, 9–12, 14–17]. But we found that most permanently or temporarily constructed houses (whether attached, semi-attached, or detached) infested with* Ae. aegypti* were more likely to be occupied by tenants rather than by owners and their families. For instance, tenement houses occupied by foreign migrant workers rather than local Thai workers are more likely to be infested with* Ae. aegypti* as they are more likely to be located in dengue transmission-prone municipal areas of Tak, Samutsakorn, Samutprakan, Chonburi, Chantaburi, Phuket, and Ranong provinces. The attached tenement houses and other tenement buildings—occupied by tenants in densely populated and impoverished areas—are often used as larval survey sites or sentinel sites for dengue vector surveillance in municipal and suburban areas. This means that unhealthy tenement houses with improper purposes—but not tenement houses themselves—create environments favorable to the breeding of* Ae. aegypti*. In practical, the local surveyors just simply use up to one hundred surveyed houses as the unit of the calculation of household index (HI): the percentage of houses infested with larvae or pupae (http://www.who.int/denguecontrol/monitoring/vector_surveillance/en/index.html). If the local surveyors consider only the numbering of surveyed houses without requiring the categories of surveyed houses, it will be likely to explain why the local authorities, as well as SAOs or even local health sectors, are likely to report always a zero-ground or HI values lower than the standard (HI < 10) despite the fact that there exist infestation levels and vulnerable numbers in dengue risk areas with unhealthy buildings and environments. Based on management by local and public health practitioners along with updated literature review, the descriptor of buildings should be reconsidered to properly reflect environmental compliance with the notification because* Ae. aegypti* is peridomestic species as it always comes close into contact with human in unhealthy buildings with improperly hygiene purposes and environments.

“*Water-holding containers*” refer to any domestic goods, apparatus, or devices that can be filled with water and applied indoor or outdoor to store the waters for drinking and other domestic uses such as washing, planting, watering, and decorating. The various water-holding containers commonly used in many households are widely distributed in most occupied buildings and surrounding environments situated in either urbanized, suburban, or rural areas of Thailand [3, 9, 10, 12, 14, 15]. Further, the potential water-holding containers are at times favorable to* Aedes* breeding. The principal water-holding containers infested with* Ae. aegypti* include (1) earthen or cement jars; (2) cement basins for bathroom or toilet; (3) plastic, fiberglass, stainless steel, or cement tanks for water storage or other applications; (4) plastic, fiberglass, or stainless steel drums or baskets; (5) earthen, plastic, glass, or metallic vessels for flowers or ornamental plants; (6) plastic or earthen flowerpots and saucers; (7) plastic or earthen bowls; and (8) earthen or ceramic pots for lotus and other aquatic plants. Such these water-holding containers are often used as the unit of the calculation of container index (CI): the percentage of water-holding containers infested with larvae or pupae (http://www.who.int/denguecontrol/monitoring/vector_surveillance/en/index.html). These potential water-holding containers, if improperly manipulated, become essential for mature gravid females of* Ae. aegypti* to oviposit on their inside wall above the water surface and then to propagate its larva offsprings. Furthermore, several water-holding plants (e.g., some genera of popularly known bromeliads such as* Aechmea*,* Neoregelia*,* Nepenthes*,* Alcantarea*, and* Billbergia*) infested with* Ae. aegypti* are commonly used in many households as ornamentals. Also, some genera (e.g.,* Musa *and* Roystonea*) whose leaf axils are filled with water are at times infested with* Ae. aegypti*. Neither is mentioned by the notification. Similar to that of the buildings, the descriptor of water-holding containers should be taken into consideration for local and public health practitioners to go the extra mile when gauging the infestation levels among larval survey sites.

“*Discarded receptacles*” refer to any combustible and incombustible matters of which the unwanted wastes become not only potentially filled with water, but also oviposited by* Ae. aegypti*. The waste disposal from any buildings or even in public spaces can reduce the oviposition and egg hatchability retention and hence curtail* Ae. aegypti* density in the community or village [3, 9, 10, 12, 14]. The following wastes—commonly found to be whether partially broken or sheared—include bowls, cups, bottles, bags, pots, jars, vessels, tyres, cans, water storage baskets or drums, coconut shells, oil tanks, planting baskets, and toys. If waste disposal is improperly manipulated by a local authority, there will be a variety of discarded receptacles as breeding places for* Ae. aegypti* if filled with water; especially if they are left outside the houses with shade environments in the community or village. Similar to that of the water-holding containers, the descriptor of discarded receptacles should be taken into particular consideration for local and public health practitioners to practice a strict regime on the search of the infestation as how the impoverished community or village effectuates more disseminated wastes.

“*Aedes breeding places*” refer to any surveyed sites or containers infested with* Ae. aegypti* whether the infestation level (HI or CI) of the breeding site is greater or lower than the designated level of dengue vector control. Focus is on the breeding site at which at least one larva or pupa of* Ae. aegypti *is found. This means that it is not only oviposited but may also produce a number of developing larvae or pupae within several days later. Considering the similar descriptors of buildings, water-holding containers, and discarded receptacles, the local and public health practitioners should take into account a number of breeding places relating to* Ae. aegypti* density in the community or village beyond vector surveillance and reporting.

Taken together, surveillance of the abundance and distribution of* Ae. aegypti* is important for dengue vector control personnel and other public health practitioners to leverage data/information needed to determine factors for dengue transmission risk or related to dengue transmission; to prioritize areas and mobilize the resources available for dengue vector control; and to implement activities and selected measures suited to reduce* Ae. aegypti *density. In this regard, dengue vector surveillance, as well as selection of appropriate surveillance strategies, is implemented by local health sectors, corroborating with the SAOs and municipalities. As for the SAO's authority, all contract SAOs not only mobilize the sufficient resources used in dengue vector control but also provide fully financial and technical support of larval survey and HI/CI report by community/village health volunteers. As with the notification, larval survey activities are likely to be implemented communitywide as they remain essential based upon the output (or outcome)/objective of the annually funded project of dengue prevention and control subsidized by the SAO.

### 3.3. Transaction of the Local Code of Abatement of* Aedes* Breeding Place

All contract SAOs that do practice a public health regulatory and legislative framework for dengue vector control arrange the written statements during which a legislative body precedes the solicitation and approval of the local code of abatement of* Aedes* breeding place whether more public clearing is deemed necessary.

*“Affirming that dengue-associated morbidity occurs in the affected people living in the administrative area of *…* Subdistrict Administrative Organization such that dengue is likely to be transmitted by Aedes vector, the *…* Subdistrict Administrative Organization has urgency in abatement of Aedes breeding place. Unless weekly water drainage of the containers or pouring chemicals is properly manipulated, the breeding places will be created as there exist not only garbage filled with water such as can, coconut shell, tyre, and other discarded matters but also waters, water*-*holding containers such as bath basins, jars, vessels, pots, and the other receptacles left in buildings or surrounding environments. As stated, this local code is arranged in regards to the section 71 along with the section 67 (paragraph 3) of the Subdistrict Council and Subdistrict Administrative Organization Act, B.E. 2537 and the section 20 of the Public Health Act, B.E. 2535.”*

This written statement on the justification of the arranged local code is articulate enough to become legislation and guide enforcement based upon the Public Health Act and the Notification of Abatement of* Aedes* Breeding Place. As to the Chapter 3 Right and Liberty of Thai People of the Constitution of the Kingdom of Thailand (2007), the arranged local code shall act radically as the conduct of the right and liberty of Thai people as issued by the sections 29, 32, 33, 34, 41, and 42. But it is possible that the written statement still needs to modify or add descriptors to fit the local code, which is not explicit and justified, or to make the communication understandable to the public audiences. The following descriptors are needed to clarify the meanings used for local and public health practitioners to operate in compliance with the local code and for the people to practice routine and proper sanitation. Other than the vertical dengue vector control, the realm of IVM approaches to vector-borne diseases offers the challenge to all LAOs to improve the efficacy, effectiveness, and sustainability of disease-vector control [3]. On the other hand, the SAOs as well as other municipalities would pay more particular attention to LDVC from where and what they get started with to what they end up with. For instance, the following disparate ideas are the alternatives they get walking.

Regarded as section 4 of the Public Health Act, every SAO has widely adopted the descriptor of “wastes” so as to dispose of them properly by selecting appropriate waste management and disposal.

*“Wastes mean any papers, clothes, foods, goods, materials, plastic bags, food holders/containers, ash, animal feces or remains, or others swept up from the street, market, animal husbandry farm, or elsewhere.” *Regarded as section 4 of Public Health Act (2002), this descriptor has been repealed after the promulgation of the Amendment Issue No. 2 of Public Health Act (2007).

*“Wastes mean any papers, clothes, foods, goods, materials, plastic bags, food holders/containers, ash, animal feces or remains, or others swept up from the street, market, animal husbandry farm, or elsewhere, as well as hazardous wastes contaminated either with biohazards or with chemical hazards from the community.”, *regarded as the section 3 of the Amendment Issue No. 2 of Public Health Act (2007).

Any unwanted matters—accordingly fallen into the term “wastes” given above—are seldom infested with* Ae. aegypti* except for plastic bags and food holders/containers which are at times filled with water. On the contrary, there are the common vectors ovipositing onto animal and plant debris and potentially transmitting the diseases. They belong to the Order Diptera (see also http://www.itis.gov/); house flies that belong to the* Musca *genus and the Muscidae family and gnat flies that belong to the* Hippelates *genus, and the Chloropidae family. Moreover, some SAOs adopted the Amendment Issue No. 2 of Public Health Act (2007) in that it emphasizes the additional phrase “*hazardous wastes contaminated either with biohazards or with chemical hazards from the community.*”It is merely meaningful to interest the people in household-level environmental management practices in effective dengue vector surveillance and control. From the perspective on IVM practices, the SAOs would modify the descriptor of “wastes” by combining the meaningful terms with respect to the sources “buildings” and the categories “water-holding containers” and “discarded receptacles”, as mentioned earlier. For instance, recently published reports from South and Southeast Asian countries demonstrated that there are some household wastes that are infested with* Ae. aegypti* or* Ae. albopictus*. In India, there are four major categories of household wastes: earthen, porcelain, and plastic containers and other coconut shells [19]. In Philippines, three major types of plastic drums (40%), metal drums (30%), and plastic containers (11%) are infested with* Ae. aegypti*, as with* Ae. albopictus* bamboo stumps (29%), plastic drums (21%), and rubber tires (19%) [20].

Apart from the descriptor of “buildings”, all the SAOs adopted the descriptor of “public spaces”, regarded as section 4 of the Public Health Act (2002).

*“Public spaces mean any public places or areas not owned by any privacies but accessible to all people exploiting or transporting.”*

**Figure 1:**
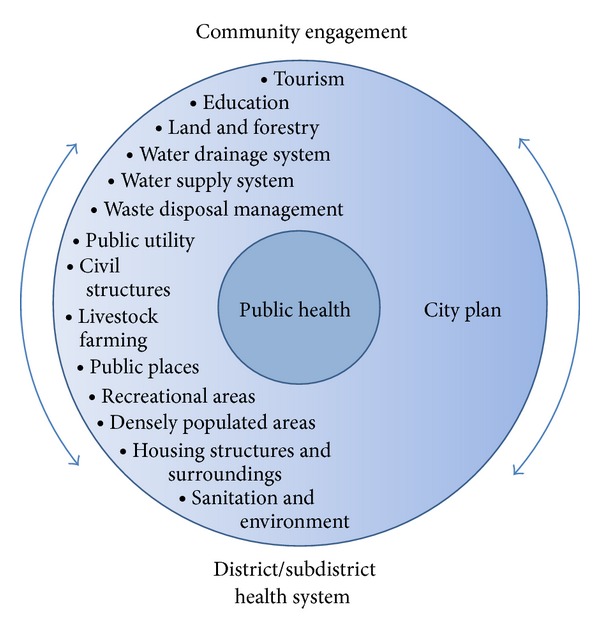
Determined missions on public health and other related firms for city development. In conjunction with district/subdistrict health system and city development, such actively engaged local governments commit to providing service delivery for public health and other human well-being to local people and community by making use of public service systems.

“Public spaces” can be defined as all opened urban areas that are indispensable for urban space and life and accessible to all members of the public in a community or society in the establishment of activities in particularly creative way. As for the city plan and development (Figure 1), the committal-to-enterprising SAOs and municipalities have become ingenious and inductive to inaugurate the exploits and benefits of urban spaces to the public. However, if there exist discarded receptacles or waste containers that are improperly disposed, the public spaces with shade environments are likely to create suitable conditions that* Ae. aegypti* can infest [9, 10, 16]. And with outdoor biting and resting behaviors,* Ae. aegypti *comes close into contact with human over space and time at which the people go for outdoor activities during the daytime. Thus, the public space is a land management strategy but where the SAOs would have disparate ideas on other effective and sustained management strategies of wastes and environments.

Section  4 of the Public Health Act (2002) issues on breeding place for disease vector. The Notification of Abatement of* Aedes* Breeding Place also issues on* Aedes *breeding place. Although the descriptor of either “*Aedes* breeding place” or “*Aedes* vector” is modified by every SAO, there does not seem to be any wrong sense left.

*“Aedes breeding places mean any conditions that create water-holding longer than 7 days as potentially risk as Aedes spp. can oviposit and further develop into larvae.”*

*“Aedes vector mean Aedes mosquito that feeds blood meal during the daytime and is commonly found during the day; also meaning its developmental stages.”*

In fact, there seem to have great variations and factors influencing both adult and immature stages development for* Ae. aegypti *and* Ae. albopictus* vectors in different local environmental conditions [15, 21–23]. For instance, as early as there seems to be any water left in water-holding containers or discarded receptacles, gravid females of* Ae. aegypti* can infest there and then. In municipal or suburban areas of Thailand, most improperly manipulated water-holding containers are infested with* Ae. aegypti* [3, 9, 10, 14–16]. In rural areas administered by the SAOs, there seems to be infested with the population dynamics of* Ae. aegypti *and* Ae. albopictus *[15]. No matter any containers infested with* Ae. aegypti* will provide baseline infestation level (CI or HI) as well as any others infested with* Ae. albopictus*. Although both species play roles in dengue and chikungunya transmission [24], the surveillance of infestation or reinfestation of* Aedes* vectors seems to show the epidemiologic implications of disease-vector control in certain dengue/chikungunya transmission risk areas.

More importantly, the regulators or authorized officials are assigned to operate in the environmental compliance of the Public Health Act and its amendment issue and notification and of the Subdistrict Council and Subdistrict Administrative Organization Act. All the SAOs adopted the descriptors of “local official” and “public health official” as mentioned earlier. Additionally, some SAOs issued the descriptor of “local government officer or employee.”

*“Local government officer or employee means any local government officer or employee whom shall be assigned, with respect to the section 44 (paragraph 2) of the Public Health Act, B.E. 2535, by local official to be imperative to execute the section 44 (paragraph 1) of that local administration on any respect or on all due respect.”*

## 4. Recommended Good Practice for SAO Administrators

No other vector-borne diseases are addressed by every SAO across the country, has dengue been put in place the priority in local health policy and planning. As mentioned earlier, every SAO has the authority to mitigate the public health burden attributed to dengue. Almost all SAOs execute fiscal policy and plans that include funded projects on dengue prevention and control because the disease can bring about sociopsychological effects on stretching the strings of popular polls and proliferating blanket coverage of integrated interventions and services to the target population and beneficiaries. Neither continuation of implementing dengue prevention and control measures and activities nor expansion of delivering health services can stop the spread of dengue unless there is pronounced change in human risk behaviors. Because dengue transmission dynamics relate urban ecology to the infestation of* Ae. aegypti* in most urban settings, it is easier to contain the environments unfavorable to the breeding of* Ae. aegypti* in the rural settings as well as to prevent risk behaviors as the foundation of the process of human behavioral change. Thus, if there is a need for the proper conduct of the local people on a large scale, the SAO should renovate local legislation and regulation properly and timely.

### 4.1. Comprehensive Legislation

For the present, the local legislation on dengue vector control adopted by the SAOs remains less comprehensive although the rules of the laws have descended to the appeal for the local applications. Regarding this, there are two topologies (A and B) of the existing local code of abatement of* Aedes* breeding place adopted by the SAOs across the country, as given below. Difference in the written statements of the local dengue vector code is dependent on the determination of applicable descriptors rather than on the determination of prescribed orders and methods, and penalties with respect to the related Public Health Acts and the ministerial regulations or notifications. Regardless of the proclamation in the Royal Thai Government Gazette, the local dengue vector control code—based on gaining the results of the local legislation evaluation along with searching its edible files available online using the Google search engine tools—is categorized into 2 premises.

- The major premise has been arranged after 2006 upon determined descriptors: (1) “Wastes”, “Buildings”, “Public spaces”, and “Public health official” prescribed by the Public Health Act (1992); (2) “Wastes” and “Local official” prescribed by the Amendment Issue No. 2 (2007); (3) “*Aedes* vector” modified from the Notification of Abatement of* Aedes* Breeding Place (2002); or (4) “Local government officer or employee” prescribed by the Public Health Act (1992).
- The minor premise has been arranged after 2008 upon determined descriptors: (1) “Wastes”, “Buildings”, “Public spaces”, and “Public health official” prescribed by the Public Health Act (1992); (2) “Wastes” and “Local official” prescribed by the Amendment Issue No. 2 (2007); or (3) “*Aedes* breeding places”, “Water-holding containers”, and “Discarded receptacles” prescribed by the Notification of Abatement of* Aedes* Breeding Place (2002).

If there are needs for local legislation on IVM for vector-borne diseases and pest control rather than on dengue vector control, the SAO administrators and legislative bodies should appreciate the meaningful descriptors and their appropriate applications because the finest descriptors will fabricate this comprehensive local legislation. Following descriptors that have epidemiological implications for a range of diseases potentially transmitted by the local vectors, including* Aedes* dengue vectors, should be considered if most local people in any rural settings are vulnerable due to human risk behaviors and environmental conditions pertaining to “Wastes,” “Water-holding containers,” “Discarded receptacles,” “Buildings,” and “Public spaces.”

More importantly, the public participation creates complex power relationships among the people or community, local health sector, and local government. Despite local legislation and regulation, the public participation usually influences the social norm as it is deemed necessary if the SAO expects strong cooperation and engagement of community stakeholders (Figure 1) [2, 11, 25]. This may be a reason why some contract SAOs are concerned about public participation and relation in addition to what they might have learned and practiced on the legislative proceedings and the dengue vector control regulation. Moreover, these SAOs facilitate the engagement of the community involvement, which is the most important practice at managing sustainable dengue vector control [11, 13, 25–27], as opposed to dengue vector control measures provided as the part of public services.

**Figure 2:**
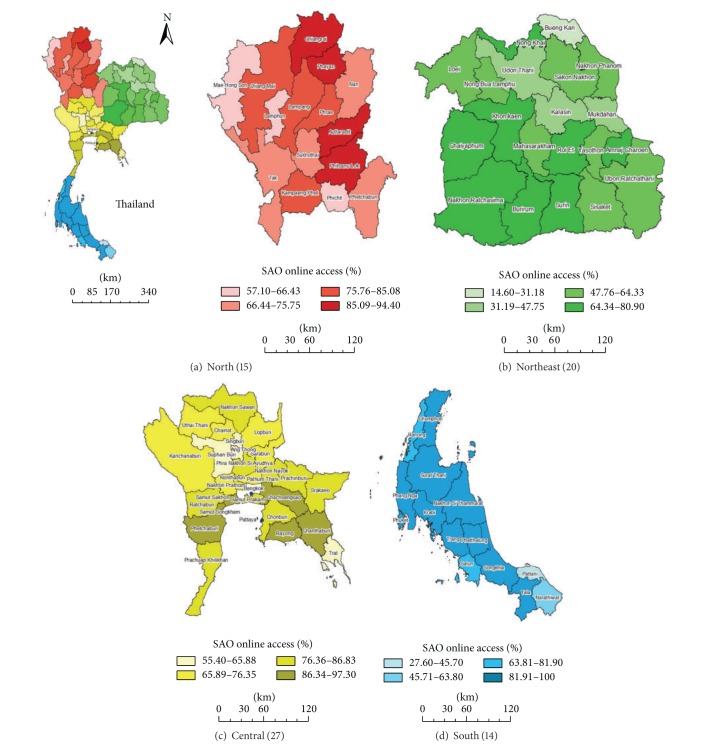
Maps on a 2013 current status of SAO websites accessible online in conjunction with the rural development in four regions (no. of provinces) of Thailand after 2011. A provincial boundary is shown for the percentage of online access in rural settings governed by the SAOs of the 76 provinces by excluding that of urban settings within the same provinces. Bangkok and Pattaya are also not included. The entire 5503 SAO databases—accessed through companion websites at http://www.tambol.com/tambol/tambolall.asp and http://www.earthpower.co.th/—include existing 5492 SAOs (Table 1) and 11 SAOs that will have been upgraded to the subdistrict municipalities. Overall percentages of online access to available SAO databases vary by region—77.4% (754/974) for the North (a), 63.0% (1356/2151) for the Northeast (b), 76.8% (1172/1526) for the Central (c), and 82.0% (699/852) for the South (d). However, none is developed for leveraging data/information required for district/subdistrict health system management or health service system management.

In addition, the information technology and better management will offer the challenges and opportunities to the SAOs in conjunction with the rural development scheme in the era of revolutionary socioeconomy and globalization. Our society is always moving to the new generation network technologies and mobile telecoms, which will enable the introduction of new messaging services and also provide communication services on mobile broadband networks. The SAOs should exploit the benefits of using the information technology (Figure 2) in leveraging data/information required for policy-making and decisions and disseminating more helpful information on the local legislation and regulation on dengue vector control to the public. If the SAO encompasses the promotion and support of collaborative governance, public participation, public services, and democracy through the systems used in the information technology and management, the local people will have increased online access to the SAO website to reach information that is necessary for their consumption. And at the same time, the SAO can use its website as an effective channel for disseminating the local legislation and regulation regarding dengue vector control, as well as for educational programs aiming at changing knowledge and practice of reducing* Aedes *breeding places.

### 4.2. Idiosyncratic Regulation

The local code of abatement of* Aedes* breeding place is designated to contain the infestation of* Aedes* vector in human habitations and then to publicly interest the people in taking action. Its meaningful enactment should approach a step-by-step contingency plan as follows.

- Having the authority issued by the local code of abatement of* Aedes* breeding place, the SAO should interest the people or public audiences in the significance of environmental management practices. Specific programs should promote motivation, readiness, and self-efficiency to conduct the desired behaviors such as cleaning the houses and surroundings, covering the water-holding containers with covers, and disposing the wastes [3, 9, 10, 12, 14, 16, 25–27]. These practices can be guided by the village health volunteers or local health personnel.
- Having human resource policy and planning, the SAO should permit the engagement of the local official(s) or the assigned local government officer(s) or employee(s) with a satisfying working environment. Determined leadership and partnership are required to leverage the community health management more than their specialty on laws, public health surveillance, or disease prevention and control. Their authority in the SAO should make protocols and tools to sustain the use of IVM approaches and dengue vector control measures [3]. A responsible local official should stipulate the existence of certain facts or problems rather than restricting or controlling the conduct of the people and prescribing the order or other methods, and should have authority to circumvent non-compliance.
- Concerning an articulate plan for effective and sustained dengue vector control, the accountable and transparent SAO engages the people or community stakeholders such that environmental management activities, or even IVM practices, and designed outcomes will be achieved effectively and sustainably [3, 9, 11, 12, 16, 26]. In case of effective and sustained dengue prevention and control, both SAO and local people and community play the pivotal role in both vertical and horizontal implementation activities through established mechanisms involved in social mobilization, support, and norm.
- Having the authority in fund infrastructure and allocation of health-related projects, the SAO mobilizes the available resources sufficient to warrant not only the obligatory implementation of dengue vector control but also the entomological survey. Routine larval surveys should monitor the abundance and distribution of* Ae. aegypti *larvae or pupae in potential containers [26] whether they are occupied or abandoned by households in human habitation areas.
- On a weekly or monthly larval survey, the SAO provides full financial and technical support for routine reporting of container and household indices by the village health volunteers. The data/information on the abundance and distribution of* Ae. aegypti* in certain infestation areas of human habitations are fundamental to monitor the effectiveness of the implementation of dengue vector control by the SAO and other enterprising counterparts. Meanwhile, these health volunteers interest the people in an effective enterprise by increasing the awareness, motivation, and involvement of the individuals and community [3, 11, 13, 25].

## Figures and Tables

**Table 1:** Current status of local administration development^a^ across the country.

Local administration organizations	2008^b^	2011^c^	2013^d^
Provincial administrative organization^e^	75	76	76
Subdistrict administrative organization^f^	6617	5693	5492
Municipality	1161	2082	2283
City	22	27	30
Town	119	155	172
Subdistrict	1020	1900	2081
Special local administration (Bangkok and Pattaya)	2	2	2

Total	7855	7853	7853

^a^Data modified from the Department of Local Administration (DLA), Ministry of Interior, http://www.dla.go.th/ and valid until ^b^15 August 2008, ^c^30 December 2011, and ^d^27 July 2013.

^b^After 2008, the data sources available for local administration organizations (LAOs) have been also deposited and online archived through other four main governmental agencies' websites: http://www.cdd.go.th/, the Community Development Department, Ministry of Interior; http://www.odloc.org/, the Office of Decentralization to the Local Government Organization Committee, Office of Permanent Secretary, Prime Minister's Office; http://thailocal.nso.go.th, the National Statistical Office of Thailand; and http://www.fpo.go.th/, the Fiscal Policy Office.

^e^The provincial administrative organization (PAO), the upper level of LAOs, covers all the districts.

^f^The subdistrict administrative organization (SAO), the lower level of LAOs, governs all the villages belonging to the subdistrict, as distinguishable of the subdistrict municipality.

**Table 2:** Milestone of provisions of the laws regarding local administration development and disease prevention and control by the SAO, 1992–2009.

Year^a^	Provision of Law^b^	Purpose of law^c^
1992	The Public Health Act, B.E. 2535	LDPC
1994	The Subdistrict Council and Subdistrict Administrative Organization Act, B.E. 2537	LAD
1995	The Subdistrict Council and Subdistrict Administrative Organization Act; The Amendment Issue No. 2, B.E. 2538	LAD
1996	The Subdistrict Council and Subdistrict Administrative Organization Act; Interior Ministerial Regulation, B.E. 2539	LAD
1997	The Constitution of the Kingdom of Thailand, B.E. 2540	LAD
1997	The Official Information Act, B.E. 2540	
1997	The Subdistrict Council and Subdistrict Administrative Organization Act; Interior Ministerial Regulation; The Amendment Issue No. 2, B.E. 2540	LAD
1998	The Subdistrict Council and Subdistrict Administrative Organization Act; Interior Ministerial Regulation; The Amendment Issue No. 3, B.E. 2541	LAD
1999	The Determining Plans and Process of Decentralization to Local Administrative Organization Act, B.E. 2542	LAD
1999	The Subdistrict Council and Subdistrict Administrative Organization Act; The Amendment Issue No. 3, B.E. 2542	LAD
2000	The Decentralization to Local Government Organization Committee Notification of a Decentralization to Local Administrative Organization Plan, B.E. 2543	LAD
2002	The Public Health Ministerial Notification of the Abatement of *Aedes* Breeding Place, B.E. 2545	LDVC
2003	The Subdistrict Council and Subdistrict Administrative Organization Act; The Amendment Issue Nos. 4 and 5, B.E. 2546	LAD
2006	The Determining Plans and Process of Decentralization to Local Administrative Organization Act; The Amendment Issue No. 2, B.E. 2549	LAD
2007	The Constitution of the Kingdom of Thailand, B.E. 2550	LAD
2007	The Public Health Act; The Amendment Issue No. 2, B.E. 2550	LDPC
2008	The Decentralization to Local Government Organization Committee Notification of a Decentralization to Local Administrative Organization Plan; The Amendment Issue No. 2, B.E. 2551	LAD
2009	The Subdistrict Council and Subdistrict Administrative Organization Act; The Amendment Issue No. 6, B.E. 2552	LAD

^a^A year (A.D.) in which the law was promulgated in the Royal Thai Government Gazette according to Buddhist Era (B.E).

^b^Valid until February 2014.

^c^Purposes of the enacted laws have been applied for the levels of governmental agencies to implement local administration development (LAD) on all due aspects and to precede local legislation on disease prevention and control (LDPC) as well as local legislation on dengue vector control (LDVC).
